# Post-Operative Adhesions: A Comprehensive Review of Mechanisms

**DOI:** 10.3390/biomedicines9080867

**Published:** 2021-07-22

**Authors:** Ali Fatehi Hassanabad, Anna N. Zarzycki, Kristina Jeon, Justin F. Deniset, Paul W. M. Fedak

**Affiliations:** 1 Section of Cardiac Surgery, Department of Cardiac Sciences, Libin Cardiovascular Institute, Cumming School of Medicine, University of Calgary, Calgary, AB T2N 2N9, Canada; ali.fatehihassanabad@ahs.ca (A.F.H.); annazarzycki00@gmail.com (A.N.Z.); jdeniset@ucalgary.ca (J.F.D.); 2 Department of Anesthesiology and Pain Medicine, Faculty of Medicine and Dentistry, University of Alberta, Edmonton, AB T6G 2R7, Canada; kjeon@ualberta.ca; 3 Department of Physiology and Pharmacology, University of Calgary, Calgary, AB T2N 1N4, Canada

**Keywords:** post-surgical adhesions, underlying mechanisms, translational research

## Abstract

Post-surgical adhesions are common in almost all surgical areas and are associated with significant rates of morbidity, mortality, and increased healthcare costs, especially when a patient requires repeat operative interventions. Many groups have studied the mechanisms driving post-surgical adhesion formation. Despite continued advancements, we are yet to identify a prevailing mechanism. It is highly likely that post-operative adhesions have a multifactorial etiology. This complex pathophysiology, coupled with our incomplete understanding of the underlying pathways, has resulted in therapeutic options that have failed to demonstrate safety and efficacy on a consistent basis. The translation of findings from basic and preclinical research into robust clinical trials has also remained elusive. Herein, we present and contextualize the latest findings surrounding mechanisms that have been implicated in post-surgical adhesion formation.

## 1. Introduction

Post-operative adhesions are pathological bonds that form between surfaces within body cavities, and range from a thin film of connective tissue to thick fibrous bridges that contain blood vessels and nerve tissue [1]. Previous publications describe post-surgical adhesions as scar tissue, whereas current understanding reflects their dynamic and regenerating nature, which can be characterized by distinct cellular and immune responses [2,3,4]. Post-operative adhesion formation following pelvic, peritoneal, and thoracic surgeries is a common response to tissue trauma and ischemia. It has been reported that pathologic adhesions develop after 95% of all operations, regardless of procedure or location in the body [5]. These fibrous bonds can directly or indirectly cause complications including severe chronic pain, organ dysfunction, and the increased need for “redo surgeries”, which may include surgeries to release the adhesions themselves (a procedure known as adhesiolysis). 

Post-surgical adhesions can prolong operative time in repeat surgeries, but can also increase the risk of mortality, as well as conferring major financial burden on the healthcare system [6,7,8,9,10,11]. The treatment of post-operative adhesions costs the US healthcare system over USD 2.5 billion annually, while complications related to post-surgical adhesions results in nearly one million additional days of inpatient care annually [12]. This staggering estimate of cost excludes expenditures such as imaging, diagnostic, and laboratory tests, ambulance and transport service, long-term morbidity costs, mental health implications, or the societal cost of early mortality [11]. Thus, there is an unmet clinical need for the development of safe and effective therapeutic options that can be used to mitigate post-operative adhesion formation. Success in such an endeavor requires a better understanding of the different mechanisms that contribute to post-surgical adhesions. Herein, we comprehensively summarize, contextualize, and critically appraise the existing data pertaining to the mechanisms that have been identified to drive post-surgical adhesion formation.

## 2. Clinical Significance of Post-Surgical Adhesions

### 2.1. Pelvic and Abdominal Adhesions

Peritoneal adhesions are commonplace after abdominal surgery, which is the most common cause of post-surgical adhesions where 70–90% of them have been attributed to a previous surgery [13,14]. It has also been shown that in 10 years following a pelvic or abdominal surgery, 35% of patients were readmitted a mean of 2.1 times for a disorder directly or potentially related to adhesions [7]. Post-surgical abdominal and pelvic adhesions also have serious clinical implications, including small bowel obstruction [15] and an increased risk profile at the time of repeat surgeries due to hemorrhage, perforation, reduced surgical exposure, and prolonged operative times [16,17,18].

### 2.2. Thoracic Adhesions

Much of the current literature on post-surgical adhesions relates to the consequences and potential prevention techniques for pelvic or peritoneal adhesions. However, post-cardiothoracic surgery adhesions are becoming more recognized as the incidence of redo surgeries and subsequent adhesion-related complications increase. Indeed, 6–17% of all coronary artery bypass and valve surgeries are reoperations [19,20]. Post-operative adhesions substantially lengthen surgical times and increase the risks of injury to the heart, lungs, and great vessels during sternal re-entry. Patients with congenital heart disease are likely to require several redo cardiac operations over their lifetime, where up to 33% of all pediatric and congenital heart surgeries are reoperations [21].

Pericardial post-surgical adhesion formation has been mainly studied in animal models [22]. Closure of the pericardium after surgery is recommended as it offers protection for the heart and great vessels during reoperation. However, this can be challenging as there is usually edematous enlargement of the intra-thoracic structures. There also exists a risk of the pericardium compressing the heart and any bypass grafts, which has led many surgeons to leave the pericardium open. Complications of intra-thoracic and cardiac adhesions include life-threatening hemorrhage during redo-sternotomy, right ventricular dysfunction, and decreased coronary artery bypass graft patency [23,24]. Repeat cardiac procedures on hearts with high levels of adhesions also increase operative time [23].

## 3. Mechanisms Driving Post-Surgical Adhesion Formation

Post-surgical adhesion formation involves three core processes: (A) inhibition of the fibrinolytic and extracellular matrix degradation systems, (B) the induction of an inflammatory response involving the production of cytokines and transforming growth factor-β (TGF-β), and (C) induction of tissue hypoxia, leading to increased expression of vascular endothelial growth factor (VEGF) [25]. From studies on peritoneal adhesions, we know the cellular components of adhesions: macrophages, eosinophils, red blood cells, tissue debris, mast cells, and fibroblasts, contained within the deposited matrix. As adhesions mature, their cell population changes: from days one to three, cells are mainly polymorphonuclear cells (PMN) leukocytes; whereas between days five to seven fibroblasts predominate [26]. Understanding how cells and factors contribute to the formation of adhesions is key for developing successful preventative strategies. Indeed, studies have aimed to elucidate key markers that are dysregulated and lead to adhesions. Factors that have been identified include those important for the regulation of inflammatory and immune responses, tissue remodeling, and angiogenesis and are briefly detailed in Table 1 and Table 2.

### 3.1. Fibrin

Cellular insult results in an outpouring of fibrin and subsequent fibrinolysis [23]. It is thought that the insult causes hypoxia and the release of reactive oxygen species (ROS) [27], which lead to inflammation and activation of the coagulation cascade where fibrin formation plays a key role [2]. Specifically, the activation of the coagulation cascade increases the production of thrombin, which is a key activator of fibrin. Fibrin production and fibrinolysis are part of the physiologic process that is necessary for tissue healing. Once the balance between fibrin production and fibrinolysis becomes compromised pathological adhesion formation occurs.

Deposition of fibrin monomers develop a polymeric matrix within which fibroblasts can adhere and produce components of extracellular matrix (ECM), setting the stage for mature adhesion formation. Within this fibrinous matrix, polymorphonuclear cells (PMN), macrophages, and mesothelial cells migrate, proliferate, and differentiate. These cells release a variety of substances including plasminogen system components, arachidonic acid metabolites, ROS, cytokines, and growth factors, which have been shown to modulate adhesion formation at different stages [28,29].

Post-operative hypoxia is common in surgical patients [30], and fibrinolysis has been shown to be decreased in hypoxic conditions [31,32,33]. Plasminogen activator inhibitors (PAIs) can prevent PAs from activating plasmin, leading to compromised fibrinolytic activity [34]. Surgery has also been shown to increase levels of PAIs [35]. In rats, tissue and plasma decreases in tPA and increases in PAI-1 have been reported to parallel the development of peritoneal adhesions [36]. The balance between fibrin deposition and degradation is critical in distinguishing between normal healing and pathological adhesion formation, where the persistence of fibrin eventually serves as a scaffold for fibroblasts and capillary in-growth, establishing more permanent connections.

### 3.2. Cellular and Immune Mechanisms

Serum-derived and soluble factors produced by platelets and inflammatory immune cells act as a network of highly active substances that initiate the healing process [29]. We know that platelets, inflammatory/immune cells, resident fibroblasts, and mesothelial cells lining body cavities are key contributors to adhesion formation. The recruitment of inflammatory and immune-related cells, such as macrophages, neutrophils, T-cells, and mast cells is facilitated by adhesions molecules and ECM components, including fibronectin and vitronectin [37]. Collectively, these cell types and the mediators they release regulate the inflammatory response, cellular migration, proliferation, angiogenesis, and tissue remodeling that controls the shift from wound healing to adhesion formation [37,38].

#### 3.2.1. The Mesothelium

The serous membranes of the peritoneal, pleural, and pericardial cavities are from a similar embryological origin and are lined with an epithelial monolayer with microvilli, also known as the mesothelium. Mesothelial cells synthesize and secrete lubricants, including glycosaminoglycans and surfactant which prevent friction and the formation of adhesions [39]. Upon insult, mesothelial cells take on a profibrotic phenotype, secreting inflammatory mediators, chemokines, growth factors, and ECM components that contribute to coagulation, fibrinolysis, and immune cell recruitment [40,41]. More recent work has demonstrated the mesothelial-to-mesenchymal transition (MMT) to be an important driver for adhesion production [42]. Pro-inflammatory cytokines TGF-β1 and IL-6 have been found to play an important role in stimulation of peritoneal MMT. Blocking IL-6 and TGF-β1 is associated with the inhibition of extracellular signal-regulated kinase (ERK) 1/2 [43]. The capability of mesothelial cells to undergo MMT following stimulation [44] positions them as a likely source of fibrogenic myofibroblasts, therefore playing important roles in adhesion formation [40].

Mesothelial cells are loosely attached to the basement membrane and can be easily detached even with slight trauma [45]. Following traumatic insult in the peritoneum, regional mesothelial cells are lost, disrupting the smooth epithelial monolayer. New mesothelial cells are recruited by a unique subset of post-operative intraperitoneal macrophages. These mesothelial cells form small islands in response to macrophage-secreted mediators and cytokines, which proliferate into sheets of mesothelial re-mesothelialization [46]. The confluence of the islands of mesothelial cells permits both large and small peritoneal injuries to heal in the same amount of time [47]. Similar to observations in the peritoneum, the detachment of pericardial mesothelial cells appears to be critical due to an observed decrease in fibrinolytic activity in areas with denuded mesothelial cells [22]. Mediators of the inflammatory response, such as TGF-β, have been reported to promote the detachment of pericardial mesothelial cells and the transition to a fibroblast phenotype, which promotes fibrosis [48]. Pericardial re-mesothelialization is hypothesized to stem from pre-existing floating pericardial mesothelial cells that remain in the pericardial cavity and activated mesothelial cells that are adjacent to the site of injury [24].

Some studies have shown that the mesothelium plays a protective role against adhesion formation. This protective function is thought to be due to the mesothelium existing as a frictionless surface [39,49,50,51,52]. However, there is continued debate on the extent to which mesothelial cells protect from adhesions, as earlier studies have disputed their preventative mechanism [53]. Reports of the protective role of the mesothelium suggest that removing the mesothelial layer is necessary for adhesion formation. Stripped and unprotected basement membrane is hypothesized to be the substrate for fibrin attachment between cell and organ surfaces, which is followed by fibroblast accumulation [39,51].

By lineage tracing, a recent study has demonstrated that an activated subset of mesothelial cells could be the origin for adhesions [54]. They identified mesothelin as a specific surface marker that is up-regulated by mesothelial cells participating in adhesion formation. The study also found these cells to be a necessary component of adhesion tissue in mice and humans. The same group has also recently identified that the main component of the adhesion cascade are cytoskeletal and calcium-regulating effectors, reporting that post-operative adhesions are triggered by calcium-dependent membrane bridges between mesothelial surfaces in the early stages after surgery [55]. These findings suggest that membrane protrusion and fusion between mesothelial surfaces may be the early event that drives post-surgical adhesion formation, while fibroblasts and immune cells likely modulate and perpetuate the adhesion response.

#### 3.2.2. Fibroblasts and Myofibroblasts

In peritoneal adhesions, the initial fibrinous exudate leads to the maintenance of cellular scaffolding, which allows the migration and seeding of fibroblast-like cells [56]. Studies have shown that the deposition of sub-peritoneal fibroblasts in the ECM is required for adhesion development [47,50,57,58,59,60]. As adhesions develop, fibroblasts continue to align and organize, causing subsequent deposition of collagen and ECM material, and establishing mature, fibrous adhesions [59,61]. Fibroblast content in murine models of adhesions has been shown to increase in the second week post-trauma, followed by the development of vessel structures and connective tissues elements [62,63]. In rabbits, adhesion development is further pronounced at 3-weeks post-injury and correlates with fibroblast content [64]. Histological analysis has also shown that fibroblasts responsible for the maturation of human peritoneal adhesions are myofibroblasts that demonstrate pronounced hyperplasia and collagen production [65]. However, these findings are mostly based on animal model and histological observations, so there remains continued debate on the early mechanisms driving adhesion formation.

Myofibroblasts are smooth muscle-like fibroblasts that express cytoskeletal proteins, such as α-smooth muscle actin (α-SMA) [66]. Fibroblasts in adhesions can develop an irreversible myofibroblast phenotype, overexpressing type I and III collagen, matrix metalloproteinase-1 (MMP-1), tissue inhibitor of metalloproteinase-1 (TIMP-1), fibronectin, VEGF, cyclooxygenase-2 (COX-2) [67] and TGF-β [68,69,70,71,72,73]. Intraperitoneal adhesions have increased α-SMA protein expression in adhesion-resident myofibroblasts, indicating increased cellular activity and a profibrotic response [74]. Other literature suggests that myofibroblast metaplasia may be a potential a source for adhesion formation [75].

Studies have identified that myofibroblast activation and ECM protein production and remodeling by fibroblasts are important mechanisms by which mild adhesions transition to dense, fibrous adhesions [22,76]. Fibroblasts and myofibroblasts secrete substantial amounts of ECM including fibronectin, hyaluronic acid, glycosaminoglycans, and proteoglycans [56]. Furthermore, myofibroblasts are stimulated by the pro-inflammatory and profibrotic growth factor TGF-β, which increases myofibroblast migration and activation [77]. Targeting the fibroblast-mediated mechanism of adhesion formation and maintenance may allow for more precise and targeted therapeutics.

#### 3.2.3. Macrophages

At 24 h post-trauma, macrophages compose the inflammatory cell population at the site of injury [78] and have been identified in long-lasting adhesions (>12 months) collected from abdominal surgery patients [79]. Conversely, a recent study has shown that although the recruitment of monocytes was induced by activated macrophages through the release of monocyte chemo-attractant protein (MCP), the number of accumulated macrophages was markedly decreased throughout the adhesive time course [41]. Nevertheless, since macrophages are capable of secreting PA, PAI, interleukin 1 (IL-1), IL-6, and tumor necrosis factor-alpha (TNFα) among others, they are thought to be fundamental in triggering adhesion formation and may play a role in adhesion maintenance [80,81,82]. Recently, PA and PAI-1 have been identified as chemo-attractant for epidermal growth factor (EGF) secreting macrophages that up-regulate the EGF receptor HER1 on peritoneal mesothelial cells in a murine model [83]. This precipitates further PAI-1 secretion, leading to decreased fibrinolysis at the site of post-operative adhesion formation [83]. Additionally, macrophages recruit more inflammatory cells to the site of injury, further progressing adhesion formation [46]. Macrophages have also been reported to recruit new mesothelial cells to the site of injury [46], and exert a direct effect by influencing mesothelial cell behavior and ECM constituents [84]. In peritoneal adhesions, resident peritoneal macrophages have been implicated as key players in the immune response that leads to adhesion formation. Notably, they have a unique autocrine activation system, whereby in response to tissue damage a chemokine (CCL1) and its receptor (CCR8) are released. Inhibition of CCL1-CCR8 interactions prevents migration of macrophages and the development of peritoneal adhesions [85].

It is important to note that the function of macrophages depends on differentiation status, which can play a role in wound healing in various tissues [86]. The M1/M2 macrophage polarization paradigm has been used to delineate roles during the pathogenesis of adhesions. M2 marker expression, associated with wound healing, tissue remodeling, ECM production, and modulation of immune and inflammatory responses to tissue injury [87,88], has shown an inverse correlation with peritoneal adhesion formation in a murine model [89]. However, the role of macrophages and their differentiation status is still under debate.

#### 3.2.4. Neutrophils

Neutrophils, the primary component of PMNs, are one of the most pronounced cell types in peritoneal cavity in the early period of peritoneal adhesion formation [45,90]. Recent observations of TGF-β+ Ly6G+ neutrophils were present in the injured serosa and submucosa at early stages of adhesion formation, indicating that neutrophils can also play a role in TGF-β secretion [91]. The production of chemokines from mesothelial and inflammatory cells, such as IL-8 and IL-1β induce the migration of neutrophils into the injured peritoneal cavity [92]. Activated mesothelial cells also directly recruit neutrophils and monocytes when subject to mechanical stress via the up-regulation of CXCL1 and MCP-1 [41].

Intraperitoneal injection of cyclophosphamide has been found to significantly reduce perioperative neutrophil counts and adhesion formation in mice [93]. Injection of polyclonal rabbit anti-rat neutrophil serum (ANS) also resulted in a reduction of neutrophil influx and significantly reduced adhesion formation in a rat model of abdominal adhesions [94]. Transient depletion of circulating neutrophils via thioglycolate-induced alteration of the immune environment has also been shown to decrease adhesion severity and burden in a murine model [41]. However, inhibition of PMN adherence has resulted in increased peritoneal adhesion formation [95,96]. A predominant product of neutrophil activity is the generation of ROS, particularly superoxide radicals. The role of oxidative stress in the pathogenesis of adhesions is well discussed in [97]. Samples of human post-operative peritoneal adhesions have demonstrated enhanced nitrotyrosine levels, commonly known as the markers of ROS [98]. Of note, the presence of ROS and ROS-sensitive transcription factor EGR-1 was also evident in a rodent peritoneal adhesion model [98]. Generally, ROS have been reported to have a direct cytotoxic effect on mesothelial cells and inhibit fibrinolysis via increasing the release of PAI-1 by mesothelial cells [94]. Treatment with superoxide dismutase (SOD) and catalase, scavengers of ROS, has been reported to significantly reduced adhesion formation in a rat model of post-operative peritoneal adhesions [94]. More recent research has shown that neutrophils within adhesion sites in mice undergo cell death and form neutrophil extracellular traps (NETs), networks of extracellular fibers primarily composed of DNA from neutrophils that contribute to adhesion pathogenesis [41]. These findings suggest that targeting neutrophils or mitigating its downstream mediators can have potentially anti-adhesive benefits. However, to avoid systemic adverse events, any such therapeutic must be precise.

#### 3.2.5. T-Cells

In a murine model of peritoneal and visceral adhesions, T-lymphocytes have been detected with highest levels on the third day after insult, suggesting a potential role of the adaptive immune system [99]. T-lymphocytes have been reported to persist in quantity and quality without regard for adhesion maturity, indicating inflammatory activity even in long-lasting adhesions [79]. Furthermore, adoptive transfer and T-cell depletion experiments have revealed that adhesion formation requires the presence of CD4+ αβ T-cells as well as the production of T-cell dependent pro-inflammatory cytokines, such as IL-17 and others [100]. In a murine model of adhesion formation, selective recruitment of Tim-3+, CCR5+, CXCR3+, and IFN-γ+ cells indicate that adhesion formation is driven by the presence of a Th1 CD4+ phenotype [101]. Mice treated with neutralizing antibodies directed against IL-16, the Th1-selective CD4+ chemo-attractant, results in significantly less adhesion formation [101]. Similar results are seen in mice genetically deficient for IFN-γ [101]. Targeting Th2 and Treg cells by pirfenidone, an anti-fibrotic agent, has also been shown to prevent peritoneal adhesions in rats [102]. The characterization of adhesions as a state of chronic inflammation suggests T-cells may play a role in signaling pathways that maintain adhesions, notably through the mediating effects of IL-6 and other cytokines [79,103]. Many fibrotic tissue disorders share a common etiology of T-cell abnormalities in host defense where it has been postulated that adhesions can fall under a similar category [104]. This further prompts consideration of adhesions as a dynamic process in remodeling tissue [79].

#### 3.2.6. Mast Cells

Mast cells were found in high concentrations in post-surgical adhesion sites [105]. As a pro-inflammatory cell, mast cells are responsible for releasing a host of factors including histamine, serotonin, cytokines, serine proteases, and chymase. Chymase, a chymotrypsin-like serine protease activates TGF-β. Mast cell-deficient mice demonstrate a significant reduction in adhesion formation [106], highlighting the importance of both mast cells and chymase in the pathophysiology of adhesions. In a post-cardiac surgery context in the hamster model, chymase inhibition has been shown to impede formation of connective tissue deposition and fibrosis [107]. Mast cells also release VEGF, an early pathogenic event that has been suggested to play a key role in adhesion formation [108].

### 3.3. Inflammation and Angiogenesis

An important feature of tissue repair and potential pathologic adhesion formation is the local inflammatory response induced by surgical trauma [109,110]. As outlined above, immune cells have been shown to play an important role in post-surgical adhesions. In this section, we focus on various inflammatory mediators that have been found to contribute to the formation and severity of post-operative adhesions. It is important to emphasize that the exact mechanisms by which these mediators drive adhesion formation is not fully delineated. A better understanding of their role in the pathogenesis of post-surgical adhesion formation will aid us in designing safer, more effective, and more precise preventative modalities.

#### 3.3.1. Interleukins and Tumor Necrosis Factor-Alpha

Interleukins (ILs) have also been implicated in adhesion development. Injured tissue releases cytokines that act individually and synergistically to induce a humoral inflammatory tissue reaction in response to insult [111]. There has been limited information on the roles of IL-6, IL-8, and IL-10 in adhesions, although they have been theorized to be a mediator of cellular response to injury [29]. Certain studies have drawn direct correlations between cytokine levels, predominately TNF-α and IL-6, and the extent of adhesion formation [112,113]. A limited human pilot study has reported high concentrations of IL-6 at 12 h and IL-1 at 48 h to be associated with adhesion reformation [114].

IL-1 has been hypothesized to promote inflammation and reduce local fibrinolytic capacity, inhibiting fibrin degradation and stimulating mesothelialization of the deposited fibrin matrix [29]. Early elevations of IL-1 has been postulated to be a reliable biological marker for post-operative adhesions [115]. IL-1 has also been shown to be at a significantly high level in the peritoneum and serum of post-operative patients [116,117], suggesting a role of IL-1 in adhesions. In human mesothelial cells, IL-1β has been shown to increase the release of PAI-1, promoting a decline in local fibrinolysis [118]. Treatment with anti-IL-1 neutralizing antibodies in a rat model of peritoneal adhesion formation served to reduce adhesion formation in comparison to controls [119]. Both IL-1 and IL-6 have been shown to stimulate mesothelial and inflammatory cells to produce and release PAI-1 and 2 in order to suppress plasminogen activation [120], suggesting a role in reduction of fibrinolytic capacity.

IL-6 is considered a marker for early tissue damage [121], and is induced by IL-1 and TNF-α in mesothelial cells in a time- and dose-dependent manner [122]. The common identification of elevated IL-6 in intra-abdominal adhesions in both patients and animal models [112,114,123] indicates the important role of lymphocyte balance in regulating ongoing inflammatory and regenerative processes that are involved in adhesion formation. Activated mesothelial cells, which can be induced by inflammation, are known to produce and secrete large amounts of IL-6 in the peritoneal cavity [124]. Previous studies have also demonstrated IL-6 to be a promoter of fibrosis via altering MMP and TIMP expression [29]. Anti-IL-6 receptor monoclonal antibody treatment in a mouse model of abdominal adhesions resulted in decreased neutrophil recruitment and adhesion formation [91].

IL-8 is secreted by mesothelial cells [125] and is a potent and highly selective chemo-attractant for neutrophils [126], but more studies are needed to elucidate its role in adhesion in formation. IL-10 is a major anti-inflammatory and immunosuppressive cytokine that modulates macrophage and lymphocyte responses to pro-inflammatory stimuli [127]. IL-10 can inhibit the secretion of pro-inflammatory mediators, such as IL-1, IL-6, IL-8, IFN-γ, and TNF-α [128]. Fibroblasts isolated from adhesions demonstrate significant increases in IL-10 in comparison to peritoneal fibroblasts [129]. In murine models of post-operative abdominal adhesions, intraperitoneal injections of exogenous IL-10 has been shown to reduce formation of adhesions [130]. However, anti-IL-10 monoclonal antibody treatment had no effect on post-operative intraperitoneal adhesion formation, nor did intraperitoneal IL-10 levels change following operation in the same murine model [131]. These findings indicate that the role of IL-10 in adhesion formation has yet to be established.

Recently, IL-17 and interferon-gamma (IFN-γ) were found in peak levels 6–12 h after abdominal surgery. Pre-stimulation of IL-17 promoted PAI-1 and inhibited tPA production, while IFN-γ enhanced this effect [132]. IFN-γ has been shown to be increased post-operatively in peritoneal fluid of mice, peaking on the fourth day [133]. In a mouse model, adhesion development was shown to depend on IFN-γ, particularly derived from natural killer T-cells [134]. IFN-γ has also been shown to induce the IL-22 receptor in vitro [134]; IL-22 expression has recently been identified to rapidly increase in the abdominal cavity 12 hours after surgery [134]. As a potential stimulatory factor, some studies have shown that tumor necrosis factor-alpha (TNF-a) has been found in abundance in post-surgical peritoneal fluid and resulting in increased production of interleukins [113,119,135].

#### 3.3.2. Transforming Growth Factor-Beta

Transforming growth factor-beta (TGF-β) is a highly studied cytokine in the context of adhesion pathophysiology. TGF-β is known to play many roles in modulating cellular behavior and the cellular environment: it is chemotactic for neutrophils, T-cells, monocytes, and fibroblasts; it induces the ECM production of various proteins, such as fibronectin, GAGs, and collagens; and it inhibits matrix degradation by altering the ratio of proteases to protease inhibitors [136]. TGF-β is hypothesized to be a principal profibrotic mediator of adhesion development. It has been shown to be co-released and work in parallel with chymase [107], as well as form local alterations of MMPs and TIMPs [38]. Mesothelial cells and adhesion fibroblasts are major sites of TGF-β expression [37], and elevated levels of TGF-β have been observed in adhesion tissues [137,138,139], in the peritoneal fluid of patients with adhesions [138,139], as well as in surgically induced adhesions in animal models [140,141]. Animal studies have shown that administration of TGF-β to the post-operative abdomen enhances formation of adhesions [77], while a blockade of TGF-β1 via inhibitory antibody treatment markedly reduces the incidence of adhesion formation [60]. An oral TGF-β type I receptor kinase inhibitor (EW-7197) has been reported to reduce incidence, quality, and tenacity of peritoneal adhesions in a dose-dependent manner via inhibition of TGF-β/Smad2/3 signaling and MMT [142]. A more recent and detailed study has reported that some of the protective anti-adhesive mechanisms of EW-7197 include a down-regulation of pro-inflammatory cytokines, attenuation of pro-inflammatory cell infiltration, inhibition of oxidative stress, decrease in excessive collagen deposition, and suppression of profibrotic genes at the site of surgery [143]. TGF-β levels post-abdominal surgery has been shown to peak at 2 h and again between 72–96 h. This correlates with the finding that TGF-β directly stimulates fibroblasts [132]. Based on these observations, it is highly likely that TGF-β is involved in adhesion maturation in later stages post-insult.

#### 3.3.3. Vascular Endothelial Growth Factor A

Angiogenesis has been hypothesized to have a critical role in adhesion formation, although the mechanisms are not clearly understood. Vascular endothelial growth factor A (VEGF-A) is a potent member of the angiogenic factor family, and involved in coagulation, fibrinolysis, and angiogenesis [144,145]. In this context, VEGF has been reported to play a pivotal role in adhesion formation as it increases vascular permeability and promotes the deposition of the fibrin-rich matrix required for subsequent cell migration and proliferation [146]. This may ultimately lead to post-operative VEGF surges [147].

VEGF has been suggested as a key mediator in adhesion formation due to the presence of VEGF in endothelial cells of blood vessels supplying pelvic adhesions [148]. Studies also suggest a role for VEGF in adhesion formation as anti-VEGF antibody administration showed a marked reduction in adhesion formation in an open surgery murine model [149,150]. Bevacizumab, a recombinant humanized anti-VEGF antibody, has been shown to reduce pericardial adhesions through selectively exerting effects on CD-31 expression with no difference in inflammatory response, ultimately preventing leukocyte emigration and angiogenesis [151].

#### 3.3.4. Matrix Metalloproteinases (MMPs)

Matrix metalloproteinases (MMPs) are constituents of the ECM involved in matrix activation and remodeling, cellular activation, and debridement of devitalized tissues. Several studies have demonstrated post-operative shifts in the ratios of various MMPs to their antagonists, the tissue inhibitors of matrix metalloproteinases (TIMPs) [152,153,154]. Serum MMP-9 and MMP-2 have been proposed as prognostic markers to identify post-operative adhesion formation [155]. Interestingly, gene therapy introducing catalytically inactive mutant MMP-9 via intraperitoneal instillation demonstrated a reduction in severity of post-operative peritoneal adhesions in a rat model [156]. Treatment of rat model of abdominal adhesions with berberine resulted in the prevention of primary adhesion formation via activation of MMP-3 and MMP-8 by directly blocking TIMP-1 activation core [157]. Alternations in MMP/TIMP ratios in the immediate post-operative period represent a decrease in enzymatic activity, where a chronic suppression leads to adhesions [153,154,158]. The change in ratios also suggests that proteolytic effects of MMPs, including degradation of matrix components, activation of growth factors, or the modulation of cellular migration and activity is important in adhesion prevention.

#### 3.3.5. Substance P and Serotonin

Other components and mechanisms have also been postulated to contribute to post-surgical adhesion formation. The pro-inflammatory neuropeptide substance P that binds to the neurokinin 1 receptor (NK-1R) has been suggested to promote peritoneal adhesion formation by limiting fibrinolytic activity in the post-operative peritoneum [159,160,161,162]. Peripheral serotonin has also been reported to modulate post-operative abdominal adhesion formation by regulating oxidative stress, fibrinolytic system, angiopoiesis, and TGF-β1 expression [163].

### 3.4. Hypoxia and Genetic Biomarkers

As noted above, hypoxia is central to adhesion development, as it triggers a cascade of intracellular responses involving hypoxia-inducible factors, lactate, ROS, reactive nitrogen species, and insulin-like growth factors [164]. The acute production of superoxide due to tissue hypoxia can induce fibroblasts to produce profibrotic factors such as TGF-β and type I collagen that promote adhesion formation [165]. Hypoxia has been reported to decrease apoptotic indexes and increase proliferation in adhesion fibroblasts, while inducing apoptosis in normal peritoneal fibroblasts [145]. Tissue hypoxia contributes to increased oxidative stress, leading to the generation of free radicals, precipitating DNA damage, and tissue injury [166]. Although it is still unknown which cells sense the initial insult that leads to a pathological phenotype downstream, a few studies have elucidated the gene expression changes implicated in adhesion formation [39,167,168,169,170]. Micro-array analysis has identified several hub genes, such as TNF and IL6, to be significant biomarkers in the pathogenesis of post-operative peritoneal adhesions [171]. Ongoing studies are examining the potential genetic predispositions to post-operative adhesion development [169].

## 4. Perspective: Clinical Translation and Future Work

As highlighted in this manuscript, post-operative adhesions place significant clinical and financial burdens on patients and healthcare systems worldwide. Although various pathways and etiologies have been implicated and suggested, we are yet to fully understand the mechanisms that drive the formation of these fibrous strands. It is highly likely that post-operative adhesions have a multifactorial pathophysiology. Given this complexity, we have not been able to safely and effectively treat or prevent post-surgical adhesions on a consistent basis. Groups have endeavored to target components of pathways that are thought to contribute to post-operative adhesions. These efforts have been hamstrung by a multitude of reasons. For example, some agents have been ineffective since they require dry and blood-less surfaces, which is difficult to guarantee during an operation. Other agents have led to serious infections that have limited their widespread clinical use. Finally, some strategies have not been universally adopted as they have not been user friendly. Failures aside, these attempts have highlighted two important concepts: first, post-operative adhesions are a major clinical issue; and second, addressing them requires a better understanding of their underlying multifactorial mechanisms.

Herein, we have summarized the latest literature on post-operative adhesion formation. We have examined the various physiologic factors and inflammatory mediators that have been identified to contribute to their formation. In doing so, we have aimed to contextualize findings to better inform future research. In our review of the literature, we have realized that most work in this area has been based on an over-simplification of post-operative adhesions. For instance, in vitro studies have mostly focused on just immune cells, or they have considered inflammatory mediators in isolation, or have heavily relied on the coagulation cascade as the single-most important contributor to adhesion formation. We posit that although each of these factors play a significant role, it is highly likely that there is cross talk between each of them. Moreover, various types of surgical-induced injury can probably elicit different associations between these and yet to be discovered factors. Since surgery is inherently invasive, it is also feasible to suggest that the disruption of an environment that keeps pro-adhesion formation markers in a homeostatic state leads to their pathologic formation. This concept should be better explored in preclinical animal studies. To date, to induce adhesions, most in vivo models have used strategies (such as buttons, for example) with limited clinical translational potential. Furthermore, it is not established whether wound healing and post-surgical adhesion formation have the same cellular and molecular origins. In all likelihood, they probably do. Therefore, more research is warranted to assess what drives one to be physiologically necessary, whereas the other becomes pathologic. Are there specific factors, or a combination of, that become dysregulated? Is post-operative adhesion formation the end product of an unchecked and overactive inflammatory response to different types of injury? If so, is it feasible to intervene upon this response at a specific time-point without compromising natural healing? Finally, if post-surgical adhesions are formed, is there an opportunity to treat them in a safe and targeted manner that does not result in the reversal of the healing process? These are just a few questions that should stimulate thought-experiments and discussions that can encourage the design of future research.

As summarized here, various mechanisms and factors have been identified to contribute to post-operative adhesion formation. In some cases, groups have been able to leverage specific components to facilitate clinical translation. For example, physical barriers that have been enriched with anti-inflammatory agents have been designed and used in the clinical setting to varying degrees of success. To further enhance clinical translation, it is important to consider the following. First, although a group of cells (such as macrophages) have been shown to play a role in post-surgical adhesion formation, these cells are not all the same. For instance, pericardial macrophages may have distinct functional effects compared to peritoneal macrophages. Similarly, cells in a given body cavity can possibly drive the presence, secretion, or replenishment of markers that are different from those in another space. Second, under physiologic conditions, cells and mediators are kept in a homeostatic state that effectively balances their pro- and anti-inflammatory properties. Preclinical studies are inherently limited as they cannot recapitulate these conditions. Third, perhaps a better understanding of wound healing processes can aid us in addressing post-operative adhesions. Fourth, large animal models of post-operative adhesions are conspicuous in their absence. To maximize clinical application, these models should be designed for specific types of operations. Equally as important, to reduce heterogeneity in their reporting, objective metrics and parameters that can be used to grade adhesions must be defined and standardized. Finally, given the reasons why many of the current approaches have failed, future work in this area must focus on developing agents and strategies that can facilitate precision and personalized medicine.

## 5. Conclusions

Post-surgical adhesions pose a great health and financial burden. A substantial amount of work has been dedicated to better understanding the exact mechanisms of adhesion formation in the surgical setting. Inflammation and immune mediators are known to play a central role in the development and severity of post-operative adhesions. Although our understanding of some of the underlying mechanisms driving adhesion formation has significantly improved over the past two decades, literature has yet to fully explain the pathogenesis and etiology of post-surgical adhesions. To further bridge the translational gaps more rigorous preclinical studies are needed to closely examine the interactions between immune cells and mediators and post-surgical adhesion formation. To facilitate precision and personalized medicine, future therapeutic strategies should be designed to directly target specific markers.

## Figures and Tables

**Table 1:** Cellular mediators and their role in adhesion formation.

Cellular Target	*Role in Adhesions*
Mesothelial Cells	Loss of protective mesothelium * Activated mesothelial cells * Profibrotic phenotype upon traumatic insult MMT may be a source of adhesiogenic myofibroblasts Secrete inflammatory mediators, chemokines, growth factors, and ECM components Directly recruit neutrophils and monocytes under mechanical stress Inflammatory mediators promote detachment of mesothelial cells ↑ surface marker mesothelin Cytoskeletal and calcium-dependent membrane bridges may drive early adhesion formation
Fibroblasts	↑ in adhesions correlated with development of vessel structures and long-lasting connective tissue elements Acquisition of myofibroblast phenotype Secretion of ECM components
Myofibroblasts	Stimulated by profibrotic TGF-β ↑ α-SMA expression indicating increased cellular activity and profibrotic response ↑ expression of type I and II collagen, MMP-1, TIMP-1, fibronectin, COX-2, and TGF-β Hyperplasia Myofibroblast activation may facilitate transition to dense fibrous adhesions
Macrophages	Predominant cell population after surgical trauma Unique autocrine activation in response to tissue damage Identified in long-lasting adhesions Recruitment of inflammatory cells Secretion of PA, PAI, IL-1, IL-6, and TNFα Recruitment of mesothelial cells to injury M2 macrophage response inversely correlated with peritoneal adhesion formation
Neutrophils	Predominant cell population in early period of adhesion formation Respond to mesothelial and inflammatory cell Release IL-8 and IL-1β Role in TGF-β secretion Generation of ROS NET formation contributes to pathogenesis Reduction or depletion has beneficial effects for reduction of adhesion formation * Inhibition increases adhesion formation *
T-Cells	Predominant cell population on third day after insult Persist in quality and quantity in mature adhesions Fibrotic disorders share T-cell abnormalities Adhesion formation requires of CD4+ αβ T-cells Production of IL-17, IL-6 IFN-γ and T-bet drives adhesion formation * Reduction of Th2 and Treg drives adhesion formation * Th1 CD4+ phenotype drives adhesion formation *
Mast Cells	High concentrations in adhesion sites Release histamine, serotonin, cytokines, serine proteases, chymase, and VEGF Deficient mice demonstrate adhesions

α-SMA = α-smooth muscle actin, COX-2 = cyclooxygenase-2, ECM = extracellular matrix, IL = interleukin, MCP = monocyte chemo-attractant protein, MMP-1 = matrix metalloproteinase-1, MMT = mesothelial-to-mesenchymal transitions, NET = neutrophil extracellular traps, ROS = reactive oxygen species, TIMP-1 = tissue inhibitor of metalloproteinase-1, TGF-β = transforming growth factor-beta, TNFα = tumor necrosis factor-alpha, VEGF = vascular endothelial growth factor, ↑ = increases, * = currently debated in the literature.

**Table 2:** Soluble mediators and their role in adhesion formation.

Signaling Molecule	Brief Role in Adhesions
Fibrin	Disruption of fibrin production and fibrinolysis homeostasis Formation of polymeric matrix for cellular adhesion and ECM production Scaffold for fibroblasts and capillary formation ↑ plasminogen activation
Tissue plasminogen activator (tPA)	Regulation of fibrinolytic system ↓ plasma levels in rat peritoneal adhesion model
Plasminogen activator inhibitor (PAI)	Inhibit tissue plasminogen activator, ↓ fibrinolysis ↑ plasma levels in rat peritoneal adhesion model
Reactive Oxygen Species (ROS)	Enhanced in peritoneal adhesion Direct cytotoxic effect on mesothelial cells Inhibit fibrinolysis through PAI-1 release in mesothelial cells Treatment by ROS scavengers reduces adhesion formation
Chymase	Activates profibrotic TGF-β
IL-6	Marker for early tissue damage Promotes fibrosis via MMP and TIMP expression ↑ in intra-abdominal adhesion models ↑ in human adhesions 12 h associated with reformation ↑ directly correlated with extent of adhesion formation Inhibition in mouse model ↓ adhesion formation Stimulate mesothelial and inflammatory cells to ↑ PAI-1/2
IL-8	Neutrophil chemo-attractant
IL-10	Modulates macrophage and lymphocyte responses Inhibit secretion of pro-inflammatory mediators ↑ in adhesion fibroblasts
IL-1	Early elevations are biological marker ↑ IL-1 in human adhesions at 48 h ↓ local fibrinolytic capacity IL-1β increases release of PAI-1 ↓ fibrinolysis
IL-17	Peak levels 6–12 h after surgery ↑ IL-17 ↑ PAI-1 and ↓ tPA
IFN-γ	Necessary for adhesion development ↑ PAI-1 and ↓ tPA
TNF-α	↑ directly correlated with extent of adhesion formation ↑ IL production
TGF-β	Principal fibrotic mediator ↑ in adhesion tissue, peritoneal fluid, and animal models ↓ reduces incidence, quality, and tenacity of adhesions
VEGF	Increases vascular permeability Promotes deposition of fibrin matrix ↑ in endothelial cells in pelvic adhesions ↓ reduces adhesions
MMP and TIMP	MMP-2/9 proposed as biomarkers MMP/TIMP ratio alterations decrease enzymatic activity Inactivating MMP results in reduction of severity Blocking TIMP-1 prevents primary adhesion formation
Substance P	↓ fibrinolytic activity
Serotonin	↑ inflammation, oxidative stress, angiopoiesis ↑ TGF-β expression Regulation of fibrinolytic system

ECM = extracellular matrix, IFN-γ = interferon-gamma, IL = interleukin, PAI = plasminogen activator inhibitors, tPA = tissue plasminogen activator, ROS = reactive oxygen species, TGF-β = transforming growth factor-beta, TNFα = tumor necrosis factor-alpha, VEGF = vascular endothelial growth factor, ↑ = increases, ↓ = decreases.

## Data Availability

This Review Article presents data from peer-reviewed publications indexed in MedLine PubMed.
